# High Frequency of Germline *TP53* Mutations in a Prospective Adult-Onset Sarcoma Cohort

**DOI:** 10.1371/journal.pone.0069026

**Published:** 2013-07-22

**Authors:** Gillian Mitchell, Mandy L. Ballinger, Stephen Wong, Chelsee Hewitt, Paul James, Mary-Anne Young, Arcadi Cipponi, Tiffany Pang, David L. Goode, Alex Dobrovic, David M. Thomas, on behalf of the International Sarcoma Kindred Study

**Affiliations:** 1 Department of Cancer Medicine, Peter MacCallum Cancer Centre, Melbourne, Victoria, Australia; 2 Research Division, Peter MacCallum Cancer Centre, Melbourne, Victoria, Australia; 3 Department of Pathology, Peter MacCallum Cancer Centre, Melbourne, Victoria, Australia; 4 Sir Peter MacCallum Department of Oncology, University of Melbourne, Melbourne, Victoria, Australia; 5 Department of Pathology, University of Melbourne, Melbourne, Victoria, Australia; Oslo University Hospital, Norway

## Abstract

Sarcomas are a key feature of Li-Fraumeni and related syndromes (LFS/LFL), associated with germline *TP53* mutations. Current penetrance estimates for *TP53* mutations are subject to significant ascertainment bias. The International Sarcoma Kindred Study is a clinic-based, prospective cohort of adult-onset sarcoma cases, without regard to family history. The entire cohort was screened for mutations in *TP53* using high-resolution melting analysis and Sanger sequencing, and multiplex-ligation-dependent probe amplification and targeted massively parallel sequencing for copy number changes. Pathogenic *TP53* mutations were detected in blood DNA of 20/559 sarcoma probands (3.6%); 17 were germline and 3 appeared to be somatically acquired. Of the germline carriers, one appeared to be mosaic, detectable in the tumor and blood, but not epithelial tissues. Germline mutation carriers were more likely to have multiple cancers (47% vs 15% for non-carriers, *P* = 3.0×10^−3^), and earlier cancer onset (33 vs 48 years, *P* = 1.19×10^−3^). The median survival of mutation carriers following first cancer diagnosis was not significantly different from non-carriers. Only 10/17 (59%) pedigrees met classical or Chompret criteria for LFS. In summary, germline *TP53* mutations are not rare in adult patients with sarcoma, with implications for screening, surveillance, treatment and genetic counselling of carriers and family members.

## Introduction

Germline *TP53* mutations result in the classical Li-Fraumeni or Li-Fraumeni-like syndromes (LFS/LFL) [1], rare inherited syndromes with a lifetime cancer penetrance up to 73% for males and ∼100% for females[2]–[7]. Historically there has been little enthusiasm in the medical community for germline *TP53* testing in LFS/LFL. Reasons include the perceptions of rarity, a lack of proven risk management strategies, and the potential for psychological harm by identifying people with an unmodifiable, extreme cancer risk [8]. However, developments in breast and whole body MRI screening [9], [10], pre-implantation genetic diagnosis for family planning, and the use of genetic information to guide cancer therapy, may influence decision-making for *TP53* genetic testing.

Mutation frequencies and penetrance estimates are largely derived from pedigree-ascertained pediatric, cohorts[4]–[6], [8], [11], fraught with ascertainment biases. Moreover, studies of LFS-associated cancers[12]–[16] suggest many germline *TP53* mutation carriers have little family history, or will be increasingly identified through genomic screens of cancer populations unselected for family history. Accurate risk counselling to the carriers identified in these ways will require study of the impact of *TP53* mutations outside of familial settings.

Sarcomas are the most common cancer type seen in LFS [17]; approximately 90% of sarcomas occur in adults [18]. To determine the incidence and clinical spectrum of germline *TP53* mutations in adult-onset sarcoma populations, a systematic screen using multiplexed ligation-dependent probe amplification and Sanger sequencing was undertaken in 559 probands consecutively recruited from adult sarcoma clinics–agnostic to family history–on the Australian arm of the International Sarcoma Kindred Study (ISKS; http://www.anzctr.org.au; http://www.australiansarcomagroup.org/sarcomakindredstudy/index.html).

## Results

Pathogenic or putatively pathogenic *TP53* events occurred in the peripheral blood DNA of 20/559 probands (3.6%), comprising 18 single nucleotide mutations or indels, and 2 whole gene deletions (Table 1). Pathogenicity was assigned as described in the Materials and Methods, and in Figure S1. Most were previously reported somatically [19], but 10 are reported here for the first time in the germline. Six variants were regarded as putatively pathogenic. The age of sarcoma onset in individuals carrying pathogenic variants was not significantly different from those carrying putatively pathogenic variants (mean±standard deviation: 38±17 years versus 40±19 years, compared to 48±18 years for non-carriers in the ISKS cohort). Seventeen were putative germline events, with the mutant allele also detected in tumor DNA and 8 tumors also demonstrating loss of heterozygosity. The remaining 3 cases suggested somatic origin: Case 18 had clinical evidence of myelodysplasia (MDS), and neither parent carried the *TP53* mutation. Both cases 19 and 20 demonstrated heterozygous whole gene deletion. While neither cases 19 or 20 had clinical evidence of MDS, both cases 18 and 19 showed widespread copy number changes in the peripheral blood–including the *RB1* locus in case 19–suggestive of somatic tumor changes rather than germline events. Only case 18 had been exposed to chemotherapy prior to blood sampling.

**Table 1:** Genetic events detected in the peripheral blood of ISKS probands.

Case	Genetic variant *TP53*	Amino acid change	Mutation type	Condel^23,24^	Reported somatic cases^22^ *	Reported germline cases^22^ *	Mutant allele present in tumour	Heterozygosity in tumour	Pathogenic
*Putative germline*									
1	c.72del	p.Lys24AsnfsX20	frameshift	NA	0	0	yes	LOH	yes
2	c.586C>T	p.Arg196X	nonsense	NA	241	13	yes	LOH	yes
3	c.559+1G>T	exon skipping	splice site	NA	10	0	yes	LOH	yes
4	c.329G>C	p.Arg110Pro	missense	del	15	0	yes	LOH	putative
5	c.783-1G>A	exon skipping	splice site	NA	7	0	yes	No LOH	yes
6	c.700T>C	p.Tyr234His	missense	del	33	0	yes	LOH	putative
7	c.853G>A	p.Glu285Lys	missense	del	186	5	yes	LOH	yes
8	c.997C>T	p.Arg333Cys	missense	del	0	0	yes	unknown	putative
9	c.473G>A	p.Arg158His	missense	del	113	9	yes	LOH	yes
10	c.877G>T	p.Gly293Trp	missense	del	6	2	yes	LOH	yes
11	c.847C>T	p.Arg283Cys	missense	del	29	10	yes	No LOH	yes
12	c.586C>T	p.Arg196X	nonsense	NA	241	13	yes	No LOH	yes
13	c.826_840del	p.Ala276_Arg280del	frameshift	NA	1	0	unknown	unknown	yes
14	c.843C>A	p.Asp281Glu	missense	del	28	0	yes	No LOH	putative
15	c.841G>A	p.Asp281Asn	missense	del	37	4	yes	No LOH	yes
16	c.469G>A	p.Val157Ile	missense	del/neut	19	0	yes	No LOH	putative
17	c.835G>A	p.Gly279Arg	missense	del	8	0	yes	No LOH	putative
*Putative somatic*									
18	c.532C>G	p.His178Asp	missense	del	9	0	yes	LOH	yes
19	whole gene del	-	deletion	NA	0	1	-	-	yes
20	whole gene del	-	deletion	NA	0	1	-	-	yes

* Database version R16 November, 2012; del: deleterious; neut: neutral; FS: frame shift; LOH: loss of heterozygosity; NA: not applicable. The assignment of pathogenicity was performed as outlined in supplementary Figure 3.

**Figure 1:**
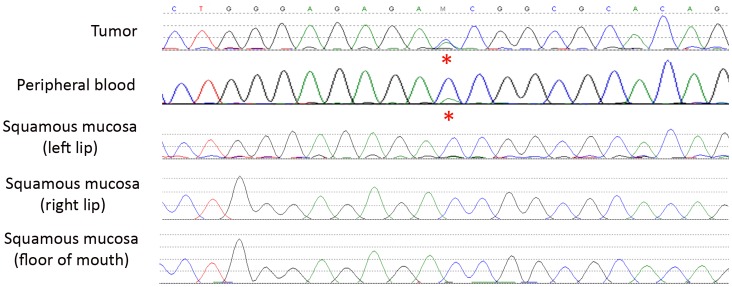
Somatic mosaicism demonstrated in an ISKS proband. Case 14 presented with an osteosarcoma of the mandible at age 19yrs. HRM analysis of the peripheral blood DNA estimated that 20–25% of alleles were mutant. The mutation was detected in tumour DNA and found to be heterozygous, but was absent in multiple other non-tumour tissues of the mouth.

Ten of 17 (59%) germline carriers had classical LFS or Chompret pedigrees that would have prompted genetic testing (Table 2). Case 14 showed somatic mosaicism, with 20–25% of mutant alleles (estimated by both Sanger sequencing and HRM analysis) in the peripheral blood, heterozygosity in the tumor, and absent in adjacent buccal mucosa (Figure 1). Somatic mosaicism for *TP53* mutations has been previously reported [20]. Of the putatively pathogenic variants, 1 occurred in a family fitting classical Li-Fraumeni criteria (case 4); two occurred in individuals fitting Chompret criteria (cases 6 & 8), and three did not demonstrate an unusual family history of cancer (cases 14, 16 & 17). This pattern is not meaningfully different than carriers of pathogenic variants.

**Table 2:** Proband cancers and clinical classification.

Case	Sex	Proband primary cancers, age at diagnosis (yrs)	Clinical classification
*Putative germline*			
1	M	**rhabdomyosarcoma 33**	LFS
2	M	**osteosarcoma 20**	LFS
3	M	**chondrosarcoma 24; liposarcoma 39**	LFS
4	M	**sarcoma NOS 37; liposarcoma 44**	LFS
5	F	**angiosarcoma 25**	Chomp LFL
6	F	breast 33; **leiomyosarcoma 48**	Chomp LFL
7	F	breast 38; **leiomyosarcoma 45**; thyroid 46	Chomp LFL
8	F	ALL 10; **Ewing sarcoma 16**	Chomp LFL
9	F	breast 26; **sarcoma NOS 36**; pheochromocytoma 37	Chomp LFL
10	M	Hodgkin’s lymphoma 34; melanoma 47; **sarcoma NOS 60**	Chomp LFL
11	M	**DSRCT 21**	Negative
12	M	testis 36; rectum 69; **leiomyosarcoma 69**	Negative
13	F	**chondrosarcoma 57**	Negative
14	M	**osteosarcoma 19**	Negative
15	M	**osteosarcoma 31**	Negative
16	F	**leiomyosarcoma 58**	Negative
17	F	**liposarcoma 62**	Negative
*Putative somatic*			
18	M	**mediastinal GCT with rhabdomyosarcomatous differentiation 19**	Chomp LFL
19	M	**GIST 65;** melanoma 69; **sarcoma NOS 76;** mycosis fungoides 76	Negative
20	F	**sarcoma NOS 80**	Negative

ALL, acute lymphoblastic leukemia; DSRCT, desmoplastic small round cell tumour; GCT, germ cell tumour; GIST, gastrointestinal stromal tumour; Chomp, Chompret; M, male; F, female.

**Figure 2:**
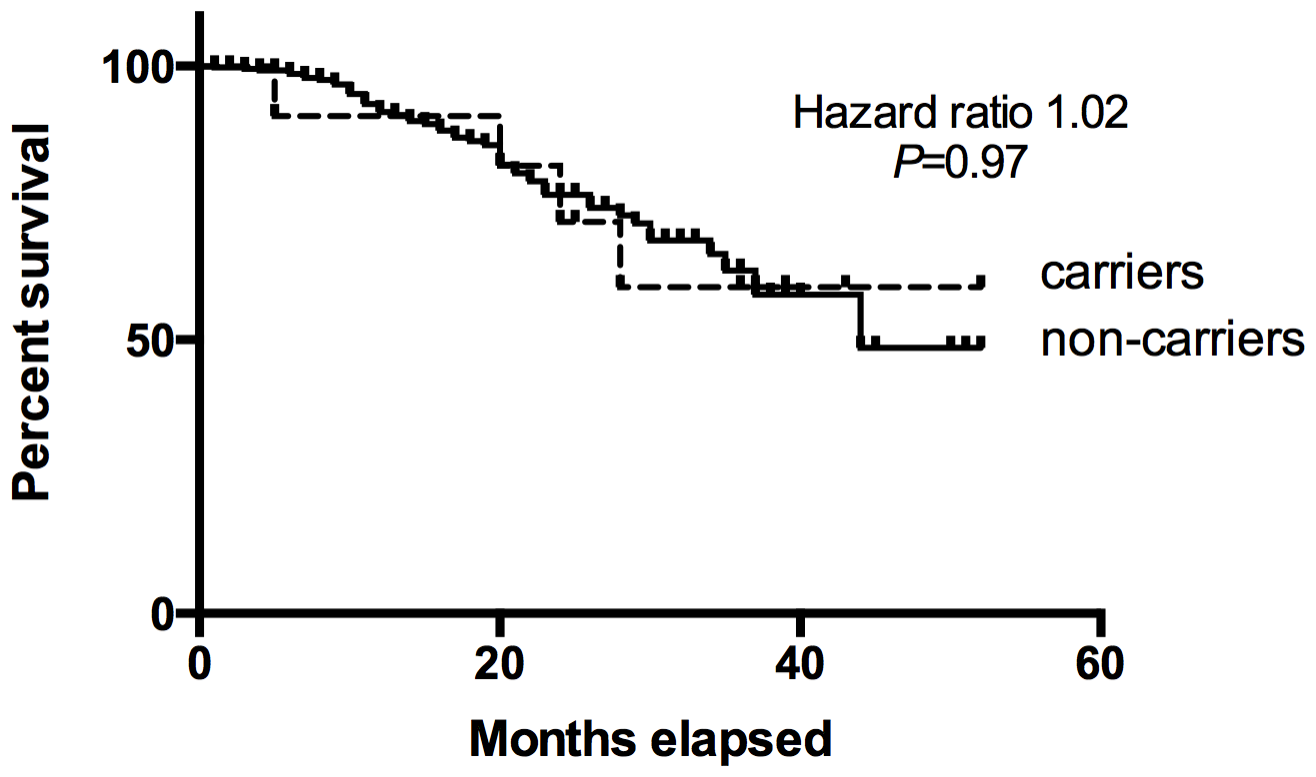
Kaplan-Meier overall survival analysis comparing *TP53* mutation carriers to non-carriers. To correct for survival bias, this analysis was limited to ISKS participants prospectively recruited from 2007 onwards (*TP53* mutation carriers, n = 11; Non-carriers, n = 420).

Regardless of family history, carriers of *TP53* mutations appeared at increased personal risk for cancer. The median age of onset (±standard deviation) of first cancer in the germline *TP53* probands was 33±14yrs compared to 48±18yrs in non-carriers (Student’s unpaired 2-tailed *t-*test *P* = 1.19×10^−3^), and the median age at first sarcoma was 36±17yrs vs 50±18yrs (*P* = 4.33×10^−3^) (Table 3). Eight of 17 mutation carriers had multiple primary cancers (Table 2), three occurring within prior radiation fields. Mutation carriers had an increased incidence of multiple cancers (47% versus 15% of non-carriers, Fisher’s exact test 2-tailed *P* = 3.0×10^−3^). With a short median follow up of 20 months, the survival of carriers in the ISKS cohort from first cancer diagnosis was not significantly different from non-carriers (Hazard ratio 1.175, 95% CI 0.44–3.12, Mantel-Cox *P* = 0.75). To investigate a possible survival bias, we also analysed those cases newly diagnosed with sarcoma during the period of recruitment from 2007 onwards (Figure 2). Again, the survival of carriers again was not significantly different to non-carriers (Hazard ratio 1.02, 95% CI 0.36–2.89, Mantel-Cox *P* = 0.97). Excluding probands, the median age at first cancer onset in *TP53* mutation positive families was 54±22yrs versus 60±18yrs in *TP53* negative families (*P* = 9.3×10^−3^). As recently reported in the IARC *TP53* database [18], more leiomyosarcoma and undifferentiated pleomorphic sarcomas were seen compared to previous reports in pediatric populations [3], [21].

**Table 3:** Characteristics of the ISKS cohort.

	Probands	
	TP53	non-TP53
n	17	539
**Gender**		
Male	9 (53%)	291 (54%)
Female	8 (47%)	248 (46%)
**Median age at diagnosis (yrs**±**SD)**		
First cancer	33±14	48±18
Range	(10–59)	(3–93)
Sarcoma	36±17	50±18
Range	(16–68)	(3–93)
**Individuals with multiple primaries**	8 (47%)	83 (15%)
**Number of tumours/individual (mean**±**SD)**	2.5±0.5	2.2±0.5
**Sarcoma subtypes**		
n	19*	546*
**Bone**		
Osteosarcoma	3 (16%)	52 (10%)
Chondrosarcoma	2 (11%)	48 (9%)
Ewing/Primitive neuroectodermal tumour	1 (5%)	47 (9%)
Other	–	3 (1%)
**Soft tissue**		
Undifferentiated pleomorphic sarcoma	3 (16%)	67 (12%)
Leiomyosarcoma	4 (21%)	61 (11%)
Fibromyxosarcoma	–	51 (9%)
Well differentiated/Dedifferentiate LPS	1 (5%)	43 (8%)
Myxoid LPS	–	27 (5%)
LPS not otherwise specified	2 (11%)	14 (3%)
Synovial sarcoma	–	38 (7%)
Angiosarcoma	1 (5%)	8 (1%)
Epithelioid sarcoma	–	8 (1%)
Malignant peripheral nerve sheath tumour	–	8 (1%)
Rhabdomyosarcoma	1 (5%)	7 (1%)
Desmoplastic small round cell tumour	1 (5%)	–
Other	–	64 (12%)
**Family history**		
Classic Li Fraumeni Syndrome	4 (24%)	4 (<1%)
Chompret Li Fraumeni Like	6 (35%)	44 (8%)
Other familial cancer syndrome	–	8 (1%)
No family history	7 (41%)	458 (85%)
Uninformative	–	25 (5%)

* some probands have >1 sarcoma; SD, standard deviation; LPS, liposarcoma.

## Discussion

The germline mutation rate observed in the ISKS cohort (3%) matches the 2–4% [12] ^,^ [21] in childhood osteosarcoma, 2–3% reported in early onset breast cancer [15], [22], but is less than reported for choroid plexus carcinomas (44%) [14]. Only 60% of carriers had a family history potentially recognisable as associated with germline *TP53* mutations. One mutation is clearly due to somatic mosaicism. Assuming a 20% new mutation rate [3], [6], the penetrance of the remaining mutations may account for the lack of a strong family history [3].

The identification of somatic and mosaic mutations remind us that peripheral blood is only a surrogate for the germline. Somatic *TP53* mutations are common in hematologic cancers, and cancer-prone individuals may harbour preclinical genetic evidence of dysplasia even in apparently normal blood. This differentiates *TP53* testing from genes such as *BRCA1/*2 or the mismatch repair genes, which appear rarely somatically mutated in hematologic malignancy. Prior mutagen exposure, including chemotherapy, may be important. It is clinically important to confirm the presence of putative germline *TP53* mutations in more than one tissue, including the tumor tissue.

These results challenge nihilistic perceptions regarding germline *TP53* mutation incidence, cancer risk and survival [23], [24]. As genomic technologies are increasingly applied to cancer cohorts, regardless of clinical or family history, more *TP53* mutation carriers will be identified and require counselling and care from their medical supports. Interpretation of purely genotypic information is difficult using data mostly ascertained on clinical or familial criteria. While more common than expected, the outlook for *TP53* mutation carriers with sarcoma appears comparable to non-carriers, and options are emerging for cancer screening [10], family planning [25], and the selection of less carcinogenic cancer treatments. Continued research into germline *TP53* mutations is critical to understanding the impact of these mutations on cancer treatments and outcomes, and to develop effective cancer screening strategies for our patients and their families.

## Materials and Methods

### International Sarcoma Kindred Study (ISKS)

ISKS is a clinic-based prospective cohort of adult-onset sarcoma cases and families aimed to investigate the hereditary aspects of this disease. Probands (n = 559, 54% male) were consecutively recruited from 6 major sarcoma treatment centres across Australia. Probands over 14 years of age with a histologically confirmed sarcoma (64% soft tissue, 36% bone subtypes) were consented to donation and use of biospecimens and provided family history information. Medical history and treatment records were obtained for each proband where possible. All reported cancer diagnoses were independently verified by reference to the medical records, Australian and New Zealand cancer registries or death certificates. Study questionnaires containing demographic, medical, epidemiological and psychosocial information were completed, including personal history of cancer or exposure to known risk factors for sarcoma. Self-reported ethnicity was 84% Caucasian, 4% Chinese or South East Asian, 3% unknown, with the remainder from diverse ethnic backgrounds.

### Clinical Classification of Families

Pedigrees to at least second degree relatives of the proband were classified according to a recognised set of clinical criteria[1], [26]–[29]. A family history was considered positive if the classical LFS or Chompret LFS criteria were met.

### Biospecimen Processing

Anti-coagulated blood was processed using a Ficoll gradient. DNA was extracted from the nucleated cell product using QIAamp DNA blood kit (Qiagen, Germany).

After micro-dissection of tumour material from formalin fixed paraffin embedded tissue, DNA was extracted using DNeasy tissue kit (Qiagen, Germany) as described previously [30].

### *TP53* HRM Screening

High resolution melt (HRM) analysis was used to screen for mutations in exons 2–11 of the *TP53* gene. PCR and HRM were performed on the LightCycler 480 (Roche Diagnostics, Australia). The reaction mixture included 1× PCR buffer, 2.5 mM MgCl2, 200 nM of each primer, 200 µM of dNTPs, 5 µM of Syto 9 (Invitrogen, Carlsbad, CA), 0.5 U of HotStarTaq polymerase (Qiagen, Valencia, CA), 10 ng DNA and PCR grade water in a total volume of 10 µL. PCR conditions included an activation step of 15 minutes at 95°C followed by 55 cycles of 95°C for 10 seconds, annealing for 10 seconds comprising 10 cycles of a touchdown from 65 to 55°C at 1°C/cycle followed by 35 cycles at 55°C, and extension at 72°C for 30 seconds; one cycle of 95°C for 1 minute, 45°C for 1 minute and a HRM step from 72 to 95°C rising at 0.02°C per second. Primers for all exons are shown in Table S1. The primers used for HRM analysis for exons 6–8 were those published in Krypuy *et al* [31] with the exception of the exon 6 reverse primer. For both exons 4 and 5, a set of three amplicons were designed to span the coding region of each exon.

All analyses were performed in duplicate. At least five different normal controls were included in each run. Where possible, a positive control sample was included for each amplicon. Samples showing an aberrant melt profile compared to normal controls via HRM were directly sequenced from a 1/10 dilution of the HRM product using the BigDye Terminator v3.1 cycle sequencing kit (Applied Biosystems, Foster City, CA) according to the manufacturer’s instructions.

Variants were triaged for pathogenicity as follows (see Figure S1). Variants were defined as pathogenic if previously reported to be associated with Li-Fraumeni syndrome in the IARC *TP53* database (R16, accessed February 2013), or they resulted in a frameshift, premature stop codon, or affected an essential splice site. Variants were considered putatively pathogenic those variants which have either been reported somatically mutated more than 5 times in the IARC database, or which were predicted to be pathogenic by Condel, or both. In all but one case (R333C), both criteria were satisfied. R333C was included because a known pathogenic variant (R337H) is located in the same region (the tetramerization domain of *TP53*), and has been reported to result in loss of function by Kato et al [32].

### *TP53* LOH Testing

For LOH analysis, tumour and matching normal DNA were amplified and sequenced. Evidence for any LOH was only inferred if the strength of the WT allele was reduced by at least 50% on the sequencing trace in tumour DNA compared to matching normal DNA.

### Mutilplex Ligation-dependent Probe Amplification (MLPA)

Large deletions or genomic rearrangements of patients were analyzed by a commercial MLPA kit (SALSA MLPA probemix P056-B1 TP53, MRC Holland, Amsterdam, The Netherlands) according to the manufacturer’s instructions. DNA (100 ng) extracted from peripheral blood was used with a normal and positive control sample included in every assay. MLPA PCR products were separated on the ABI3730 instrument (Applied Biosystems) and peak heights for each PCR product were compared to a normal sample using GeneMarker software to determine gene dosage for each individual exon. Every positive result was repeated at least twice.

The two cases with whole gene deletion were verified by using custom exon capture using Haloplex reagents (Agilent Technologies, Inc., Santa Clara, CA). Custom capture reagents were designed including the entire gene for *TP53,* as well as coding exons for 84 additional genes including *RB1.* Copy number states for all loci were inferred from SNP allelic ratios and the CONTRA algorithm for identifying copy-number gains and losses using fluctuations in sequencing read depth [33]. Target regions were divided into 100 bp bins for the CONTRA analysis, to adjust for the limited number of target regions and deep read coverage of the custom capture.

### Statistical Analyses

Statistical analyses of age at cancer diagnosis were performed using a Student’s unpaired two-tailed t-test with unequal variance, and where appropriate a Fisher’s exact test. Survival analyses used a Log-rank (Mantel-Cox) test for survival comparisons.

### Ethics Statement

This project was conducted under the auspices of the human research ethics committee of the Peter MacCallum Cancer Centre (HREC approval number 09/11). Participants over the age of 15 gave written consent to the study. For participants below 18 years written consent was obtained from their legal guardians. For participants between 16 and 18 years, written consent was obtained from both participants as well as their legal guardians.

## Supporting Information

Figure S1 **Assignment of pathogenicity for** ***TP53*** **variants.**
(TIFF)

Table S1 ***TP53*** **HRM and Sequencing Primers.**
(XLSX)
